# The Mental Health State of Canadian Ophthalmologists during the COVID-19 Pandemic: A Survey-Based Study and Review

**DOI:** 10.3390/vision7010023

**Published:** 2023-03-17

**Authors:** Mélanie Hébert, Soumaya Bouhout, Ellen E. Freeman, Marie-Josée Aubin

**Affiliations:** 1 Department of Ophthalmology, Université Laval, Quebec City, QC G1V 0A6, Canada; 2 Department of Ophthalmology, Université de Montréal, Montreal, QC H3T 1J4, Canada; 3 School of Epidemiology and Public Health, University of Ottawa, Ottawa, ON K1G 5Z3, Canada; 4 Ottawa Hospital Research Institute, Ottawa, ON K1Y 4E9, Canada; 5 University Ophthalmology Center, Centre Intégré Universitaire de Santé et de Services Sociaux de l’Est-de-l’Île-de-Montréal—Hôpital Maisonneuve-Rosemont, Montreal, QC H1T 2M4, Canada; 6 Department of Social and Preventive Medicine, School of Public Health, Université de Montréal, Montreal, QC H3T 1J4, Canada

**Keywords:** coronavirus disease 2019, mental health, ophthalmology, anxiety, depression, post-traumatic stress disorder

## Abstract

The coronavirus disease 2019 (COVID-19) pandemic disrupted the practice of medicine, causing stress and uncertainty among ophthalmologists. This cross-sectional, survey-based study of Canadian Ophthalmological Society members (*n* = 1152) aims to report on Canadian ophthalmologists’ mental health during the COVID-19 pandemic. Four questionnaires were administered between December 2020 and May 2021: the Patient Health Questionnaire-9 (PHQ-9), Generalized Anxiety Disorder-7 (GAD-7), the 7-item Insomnia Severity Index (ISI), and the Impact of Event Scale—Revised (IES-R). From all of the responses, 60/85 answers were deemed complete and were included. The median age was 50–59 years and 53% were women. On PHQ-9, most respondents had no or minimal depressive symptoms (*n* = 38, 63%), while 12% (*n* = 7) had moderately severe depressive symptoms and 12% (*n* = 7) reported impaired daily functioning and/or thoughts of suicide or self-harm. On the GAD-7 scale, 65% (*n* = 39) had no significant anxiety, while 13% (*n* = 8) had moderate to severe anxiety. Most respondents did not have clinically significant insomnia (*n* = 41, 68%). Finally, 16 respondents (27%) had an IES-R score ≥24 suggesting possible post-traumatic stress disorder. No significant differences were found based on demographics. During the COVID-19 pandemic, up to 40% of respondents experienced varying degrees of depression, anxiety, insomnia, and distress from the event. In 12%, there were concerns for impaired daily functioning and/or suicidal thoughts.

## 1. Introduction

At the onset of the coronavirus disease 2019 (COVID-19) pandemic, the death of ophthalmologist Li Wenliang, one of the first doctors to recognize the outbreak [1], rattled the entire ophthalmology community regarding the dangers of COVID-19. Since then, the pandemic has grown to epic proportions, and due to the proximity between ophthalmologists and patients, the former are at great risk for contagion [2]. This situation placed ophthalmologists in a precarious position to continue offering care to patients at the risk of their own health and that of their loved ones. This has had an important impact on the mental health of ophthalmologists in various settings and countries, leading to significant depression, anxiety, stress, and insomnia in one-third to two-thirds of ophthalmologists abroad [3,4,5,6,7,8].

Multiple public health measures have also been implemented leading to important changes in the practice and livelihood of ophthalmologists. These include post-exposure and travel quarantine, the use of personal protective equipment and shields at the slit lamp, the closure of operating rooms and clinics, the postponement of surgeries and visits, the restriction in the number of daily patients, the use of videoconferencing and telehealth tools, and the reassignments to other clinical settings, such as long-term care homes or COVID-19 medical wards. These changes can lead to further uncertainty and novelty, which can affect the mental health of ophthalmologists.

We therefore sought to offer an overview of the state of mental health among ophthalmologists in Canada during the pandemic to provide national data to inform, destigmatize, and guide future interventions. We expected to find higher rates of anxiety, depression, stress, and insomnia among ophthalmologists in similar proportions to other countries, though this could be partially mitigated by robust infection control measures that decreased the relative number of cases nationally.

## 2. Materials and Methods

### 2.1. Study Design and Population

This is a cross-sectional, survey-based study that was approved by the Institutional Review Board of the Centre intégré universitaire de santé et de services sociaux de l’Est-de-l’Île-de-Montréal (MP-12-2021-2299) and respects the tenets of the Declaration of Helsinki. Implicit consent was obtained when participants answered the survey questions.

An anonymous survey was distributed by email to all members of the Canadian Ophthalmological Society (*n* = 1152; 516 female and 636 male) between 3 December 2020 and 22 May 2021. Two messages were sent to invite members to answer the survey, one on 3 December 2020, and a reminder on 12 April 2021. Members include trainees (*n* = 193) in ophthalmology (i.e., residents, fellows), ophthalmology attendings (*n* = 906), and retired ophthalmologists (*n* = 53). All are referred to as “ophthalmologists” unless level of training is specifically addressed. Survey data were collected and managed using Research Electronic Data Capture (REDCap) tools [9,10] hosted at the Centre intégré universitaire de santé et de services sociaux de l’Est-de-l’Île-de-Montréal—Hôpital Maisonneuve-Rosemont. Data on age category, sex, level of training (i.e., resident, fellow, attending, retired), subspecialty if applicable, city of practice, and reassignment during the pandemic if applicable were collected from respondents. Freeform responses were also accepted for additional comments regarding the impact of the COVID-19 pandemic on respondents. Answers were considered for inclusion in this study if all questions were answered on at least one of the four questionnaires or if there was one written comment.

### 2.2. Mental Health Questionnaires

To assess the mental health of ophthalmologists, a combination of questionnaires was employed, using the validated French or English version of the questionnaires according to respondent preference. The Patient Health Questionnaire-9 (PHQ-9) [11] was used to assess depressive symptoms, the Generalized Anxiety Disorder-7 Scale (GAD-7) [12] was used to assess anxiety symptoms, the 7-item Insomnia Severity Index (ISI) [13] was used to assess quality of sleep, and the Impact of Event Scale-Revised (IES-R) [14,15] was used to assess the general impact of the pandemic on respondents. Results of each mental health questionnaire are presented as a numerical score, which is the sum of each item scored in the questionnaire.

The PHQ-9 assesses depressive symptoms during the past two weeks. It is a reliable and valid tool to screen for major depressive disorder (88% sensitivity and 88% specificity with a PHQ-9 score ≥10) [11]. It ranges from 0 to 27 and includes nine questions regarding symptoms of depression that are graded as 0 (not at all), 1 (several days), 2 (more than half the days), and 3 (nearly every day). The total score can be categorized as representing none or minimal depressive symptoms from 0 to 4, mild symptoms from 5 to 9, moderate symptoms from 10 to 14, moderately severe symptoms from 15 to 19, and severe symptoms when equal or greater than 20. Another item of the questionnaire allows respondents to characterize how difficult these symptoms have rendered daily functioning, and impairment is deemed present when they respond “very difficult” or “extremely difficult”.

The GAD-7 assesses anxiety symptoms over the past two weeks. It has good sensitivity (89%) and specificity (82%) for detecting generalized anxiety disorder with a GAD-7 score ≥10 [12]. It ranges from 0 to 21 and includes seven questions on anxiety symptoms that are graded in the same way as the PHQ-9 with 0 (not at all) through 3 (nearly every day). Additionally, cutoff scores of 5 to 9, 10 to 14, and 15 to 21 represent mild, moderate, and severe anxiety, respectively. As is the case for the PHQ-9, another item is used to assess impairment in functioning when daily activities are considered “very difficult” or “extremely difficult” because of the anxiety symptoms.

The ISI is a reliable and valid measure of assessing insomnia, which correlates to sleep diaries and polysomnography [13]. Scores range from 0 to 28. A score from 0 to 7 represents no clinically significant insomnia, 8 to 14 subthreshold insomnia, 15 to 21 clinical insomnia of moderate severity, and 22 to 28 severe clinical insomnia.

Finally, the IES-R is a reliable and valid tool to assess the response to a traumatic event [15]. Scores range from 0 to 88. There are 22 items scored from 0 to 4 describing different difficulties related to a stressful life event and respondents are asked to judge whether they were affected by these in the past seven days, either “Not at all”, “A little bit”, “Moderately”, “Quite a bit”, or “Extremely”. The total scores are categorized as follows: subclinical distress (0–8), mild distress (9–25), moderate distress (26–43), and severe distress (44–88) [16]. Scores of ≥33 represent the best cutoff for probable post-traumatic stress disorder (PTSD) [17], while at ≥37, this score becomes high enough to suppress immunity even 10 years after the event [18].

The authors decided to choose these questionnaires due to their previous validity in multiple studies, their common use in psychology, and their specific use in health care workers [3,4,5,6,7,8,19,20,21,22,23,24,25,26,27].

### 2.3. Literature Review

A literature review was conducted on 3 April 2022, using the PubMed database with a combination of search terms including “mental health” AND “ophthalmology”, for papers relating to the mental health of ophthalmologists and ophthalmology trainees (i.e., fellows and residents). A total of 1119 titles were reviewed by a single investigator (MH) of which 20 titles were retained. After full text revision, 14 other survey studies and 1 review paper were included in the review.

### 2.4. Statistical Analysis

Data are presented as median (first quartile, third quartile) for continuous, non-normally distributed variables and as frequencies (percentages) for categorical variables. Characteristics and variables were compared between groups (e.g., age category, sex, region of practice, subspecialty, level of training) using Mann–Whitney U test or Kruskal–Wallis test for continuous variables and chi-square analysis or Fisher’s exact test for categorical variables, as appropriate. Shapiro–Wilk test and Q-Q plots with 95% confidence intervals were used to test for normality of distribution in continuous variables.

Statistical analyses were performed using IBM SPSS Statistics for Windows (version 25.0; IBM Corp., Armonk, NY, USA). *p*-values < 0.05 were considered statistically significant. One author (MH) had full access to all the data in the study and takes responsibility for its integrity and the data analysis.

## 3. Results

### 3.1. Respondent Baseline Characteristics

Over the study period, 85 answers were submitted through the survey form of which 60 (5%) unique answers were deemed complete enough for inclusion, including 57 that had four complete questionnaires. The 25 answers that were not included in the analysis consisted of respondents who started answering demographic questions but did not complete a questionnaire or write a freeform comment.

The median age category of respondents was 50–59 (30–39, 60–69) years, and 32 (53%) were women. Most respondents were attendings (*n* = 52, 87%) and among those who disclosed their subspecialties, most were general or comprehensive ophthalmologists (*n* = 21, 41%), glaucoma specialists (*n* = 9, 18%), or oculoplasticians (*n* = 6, 12%). Most respondents practiced either in Montreal (*n* = 14, 23%) or Quebec City (*n* = 12, 20%). There were, however, respondents from across Canada including Winnipeg (*n* = 5, 8%), Vancouver (*n* = 4, 7%), Toronto (*n* = 3, 5%), Calgary (*n* = 3, 5%), Edmonton (*n* = 2, 3%), and Charlottetown (*n* = 1, 2%). 

### 3.2. Mental Health Questionnaire Results

**Figure 1:**
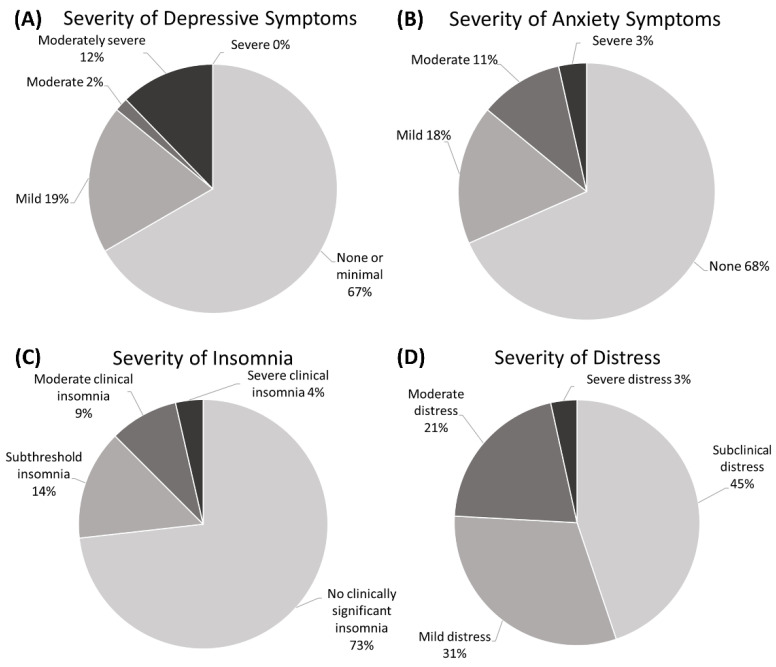
Pie charts illustrating the proportion of respondents with varying severities of mental health symptoms according to the (**A**) Patient Health Questionnaire-9, the (**B**) Generalized Anxiety Disorder-7 scale, the (**C**) Insomnia Severity Index, and the (**D**) Impact of Event Scale-Revised score.

The overall results of the mental health questionnaires’ scores are summarized in Figure 1. The median score on the PHQ-9 for depressive symptoms was 2 (0, 6.5) (range: 0–19). Most respondents (*n* = 38, 67%) had no or minimal depressive symptoms, while 11 (19%) had mild depressive symptoms, 1 (2%) had moderate depressive symptoms, and 7 (12%) had moderately severe depressive symptoms. No respondents were deemed to have severe depressive symptoms by the PHQ-9 score. However, of the 57 respondents who answered, 4 (7%) had a daily functioning considered impaired and 4 (7%) answered “Several days” to the prompt of whether they had “Thoughts that you would be better off dead or of hurting yourself in some way” over the last two weeks. Together, both statements encompassed 7 (12%) respondents.

Results for the GAD-7 scale had a median score of 2 (0,6) (range: 0–18). A majority (*n* = 39, 70%) had no significant anxiety, while 10 (18%) had mild anxiety, 6 (11%) had moderate anxiety, and 2 (4%) had severe anxiety. One respondent (2%) was deemed to have impaired functioning due to their anxiety symptoms. 

The ISI had a median of 4 (1, 8) (range: 0–24). No clinically significant insomnia was found in 41 (73%) respondents, subthreshold insomnia in 8 (14%), moderate clinical insomnia in 5 (9%), and severe clinical insomnia in 2 (4%). 

The median IES-R was 10 (4, 25.25) (range: 0–46). Subclinical distress was reported in 26 respondents (45%), mild distress in 18 (31%), moderate distress in 12 (21%), and severe distress in 2 (3%). Among these, there were 8 (14%) who had scores ≥33, indicating a probable diagnosis of PTSD [17] among whom 5 (9%) had scores ≥37 compatible with a suppression of immunity [18]. 

When comparing the mental health questionnaires’ scores among different demographic categories, there were no discernable statistically significant differences. Regarding age categories, sex, language, training level, and subspecialties, there were no differences with regards to PHQ-9 categories (age *p* = 0.49, sex *p* = 0.59, language *p* = 0.82, training *p* = 0.32, and subspecialty *p* = 0.80), GAD-7 categories (age *p* = 0.80, sex *p* = 0.44, language *p* = 0.70, training *p* = 0.55, subspecialty *p* = 0.93), ISI categories (age *p* = 0.67, sex *p* = 0.93, language *p* = 0.58, training *p* = 0.44, subspecialty *p* = 0.62), or IES-R categories (age *p* = 0.49, sex *p* = 0.97, language *p* = 0.29, training *p* = 0.57, subspecialty *p* = 0.70), respectively.

Potentially clinically significant results were, however, found regarding the reassignment of respondents. These included reassignments to non-clinical COVID-19 work, COVID-19 wards, and long-term care homes. Among respondents who had been reassigned (*n* = 6), there were 2 (33%) that had a score ≥37 on the IES-R questionnaire compared to 3 (6%) among those who were not reassigned (*p* = 0.08). On the GAD-7 questionnaire, there were more mild (*n* = 8, 16%) or moderate anxiety symptoms (*n* = 4, 8%) among those who were not reassigned compared to those who were (*n* = 3, 50% and *n* = 2, 33%, respectively) (*p* = 0.08 and *p* = 0.12, respectively). The participant who reported being impaired by anxiety symptoms had been reassigned (*p* = 0.11).

Among the 36 freeform comments, teleconsultations and virtual visits were mentioned in fifteen comments (42%). Of these, 8/15 (53%) expressed the limitations of telemedicine in ophthalmology including how patients still needed to come in for testing and/or how this could be a source of anxiety due to uncertainty of diagnosis or disease progression. On the other hand, 2/15 (13%) mentioned this was a positive addition to their practice, allowing them to mitigate the risk of COVID-19 and still provide care. The remaining 5/15 (33%) acknowledged having tried telemedicine without positive or negative comments. Furthermore, reductions in OR time and patient volume in clinics with concurrent increasing waiting lists and financial uncertainty were a recurring theme among reported answers (*n* = 18, 50%).

## 4. Discussion

The pandemic affected healthcare workers in multiple ways from disruption of their normal working lives to reassignments to new environments. Though ophthalmologists were not typically on the frontlines combatting the illness, multiple members of the specialty found their daily practice significantly changed by the pandemic, and many felt particularly at risk given the proximity to patients by the nature of the ophthalmological exam. In this study, we reported on the mental health symptoms of ophthalmologists. The results point to a dire need to address mental health in the ophthalmology community. Indeed, nearly a third of respondents had some degree of depression with 12% showing signs of moderately severe depression. Additionally, another seven respondents (12%) had impaired daily functioning and/or active or passive suicidal thoughts or thoughts of self-harm. A quarter of respondents had significant anxiety symptoms and another quarter demonstrated clinical signs of PTSD. This is very concerning but provides national data regarding the mental health of Canadian ophthalmologists and sheds light on the importance of assessing mental health among Canadian ophthalmologists at this time.

Although not statistically significant, among ophthalmologists that were reassigned to different units, there was a higher tendency for PTSD and anxiety. This could be explained by the different environments, pathologies, and care in a new setting compared to the ophthalmology clinic. This is a clinically significant finding that would require proper intervention to help ophthalmologists needing assistance. 

To the best of our knowledge, there was only one study evaluating the state of burnout and mental health in Canada prior to the pandemic where up to 35% of ophthalmologists felt a sense of psychological distress [28]. However, the use of unique scales and questionnaires in this particular study makes it challenging to compare it with our study. 

Similar studies have surveyed ophthalmologists in their respective countries during the COVID-19 pandemic and have found comparable rates of symptoms compared to this study. In China, at the start of the pandemic, a survey conducted in the ophthalmology and otolaryngology departments revealed that 52% of the ophthalmologists surveyed reported significant fatigue. Overall, 33% also reported mild depressive symptoms [3]. In Saudi Arabia, 29% of respondents had depression, 38% had anxiety, and 15% had insomnia; these significant mental health outcomes were more likely to be found in women [4]. In India, a widespread survey revealed a mean PHQ-9 of 3.98 ± 4.65 with nearly a third exhibiting some degree of depression as was the case in our study. However, only 4% showed signs of severe depression, in contrast to the 12% of moderately severe depressive symptoms found among our respondents [5]. Another Indian survey of ophthalmology healthcare workers and patients found a higher mean PHQ-9 score of 9.50 ± 4.77 among healthcare workers compared to 5.98 ± 3.49 in patients (*p* = 0.001). This corresponded to over half of healthcare workers suffering from moderate to severe depression [6]. Over half of practicing ophthalmologists in India in another survey disclosed having depression and anxiety using the Depression, Anxiety and Stress Scale-21 (DASS-21) [19]. In Turkey, ophthalmologists were surveyed using the Beck anxiety scale revealing more than a third of respondents had some degree of anxiety symptoms [20]. In another Turkish survey, 91% mentioned having anxiety regarding the pandemic, especially due to fear of transmitting the disease [21]. In another survey, there were symptoms of depression, anxiety, stress, and insomnia in 65%, 57%, 43%, and 47% of respondents, respectively [7]. In Spain, 59% of respondents had increased anxiety [8]. In New Zealand, a single study reported that ophthalmologists felt some physical health benefit with the pandemic lockdowns and did not report changes in mental health or social wellbeing, contrary to our study and the ones mentioned [22]. Overall, the proportions of symptoms found in the surveys reviewed are similar to those found in our study (Table 1).

In articles relating to the mental health of ophthalmology trainees, there was a reported significant increase in anxiety and stress ranging from 54.8% to 70% [23,24]. Mild depression was also found in a third of respondents [25], and one in five young ophthalmologists surveyed considered needing a psychological assessment and help after the pandemic [26]. Explanations for these included concerns of spreading the virus to patients or loved ones, redeployments, and interruptions in ophthalmology training [29]. 

Ophthalmology is a specialty that is in close contact with patients through the nature of the ophthalmological exam. Other specialties have also seen difficulties with addressing the need of COVID-19 patients on hospital wards. In particular, at the center of the outbreak in Wuhan, another team surveyed medical and nursing staff at the onset of the pandemic. Using the same questionnaires as those used in our study, Kang et al. (2020) clustered respondents and found that 36% had subthreshold mental health disturbances, 34.4% mild disturbances, 22.4% moderate disturbances, and 6.2% severe disturbances [16]. This is greater than what was found in our study in which more than 60% of respondents had subthreshold findings across all questionnaires. The same questionnaires were also administered by Lai et al. (2020) in multiple hospitals in China where nearly half of respondents had symptoms of depression and anxiety, a third had insomnia, and more than 70% had symptoms of distress [30]. When looking specifically at staff working in the intensive care units, almost half of respondents had symptoms suggesting probable diagnoses of PTSD, severe depression, anxiety, or problem drinking [31]. There seems to be consistency that the healthcare practitioners who were in direct contact with COVID-19 patient care generally report greater mental health symptoms [16,30,32].

Among articles that explored methods to mitigate symptoms of PTSD during the COVID-19 pandemic, some found that clinicians with more disruption were less likely to access psychological material and resources [33]. Coping mechanisms proposed in previous studies include promoting an individual positive lifestyle and mindfulness, using psychological resources, accessing digital psychological recommendations, and participating in therapeutic support groups. Institutional support of staff mental health and wellbeing is also essential. Methods include following public health measures, providing complete personal protective equipment, having regular information meetings and check-ups with the health care team, and providing psychological courses or hotline assistance. In the ophthalmology community, local to national associations have provided online resources to guide ophthalmologists, although none of the respondents in our study mentioned using resources.

A limitation of this study includes the cross-sectional design, which limits our information on the temporal relationship of COVID-19 and the onset of the mental health issues and limits the results to a specific time period in which the survey results may have been dependent on local COVID-19 rates. This was somewhat mitigated by sending reminders at the peaks of infectious waves to better reflect the impact of the pandemic on mental health. Additionally, despite two reminders to respond to the survey, there was a relatively low response rate among the membership of the Canadian Ophthalmological Society. This could be due in part to mental health stigma, which may lead to selection bias wherein respondents who had significant symptoms may either be compelled to answer the survey or avoid answering it.

## 5. Conclusions

In conclusion, during the COVID-19 pandemic, up to 40% of responding ophthalmologists reported experiencing varying degrees of depression, anxiety, insomnia, and distress from the event. This includes a staggering 12% who reported impairment due to depressive symptoms and/or thoughts of suicide or self-harm. Specific associations with demographics could not be identified, but these results suggest that an important part of the healthcare system recovery from the pandemic will need to focus its attention on helping members of the profession return to a healthy state.

## Figures and Tables

**Table 1:** Proportions of anxiety/stress, depression, and fatigue/insomnia reported among ophthalmologists and ophthalmology residents during the coronavirus disease 2019 (COVID-19) pandemic in each survey study included in the review (*n* = 14).

Study Authors	Country	# Ophthalmologists	Anxiety/Stress	Depression	Fatigue/Insomnia
[3]	China	2155 (+1757 otolaryngologists)	NA	PHQ-9 ≥ 5 in 33.2% overall	Clinically relevant fatigue (≥4/10) in 52.4%
[4]	Saudi Arabia	107 (66 residents)	GAD-7 ≥ 5 in 46.7%; GAD-7 ≥ 7 in 38.3%; PSS-10 ≥ 14 (moderate to high stress) in 71.9%	PHQ-9 ≥ 5 in 50.6%; PHQ-9 ≥ 10 in 29.0%	ISI ≥ 8 in 44.9%; ISI ≥ 15 in 15%
[5]	India	2355 (475 in training)	NA	Mean PHQ-9 3.98 ± 4.65; PHQ-9 ≥ 5 in 32.6%; PHQ-9 ≥ 10 in 11.2%	NA
[6]	India	40 HCW and 200 patients in ophthalmology	NA	Mean PHQ-9 9.50 ± 4.77 in HCW vs. 5.98 ± 3.49 in patients (*p* = 0.001); PHQ-9 ≥ 5 in 80% and PHQ-9 ≥ 10 in 52.5% of HCW	NA
[19]	India	144	DASS-A ≥ 8 in 51.4%; DASS-S ≥ 15 in 13.9%	DASS-D ≥ 10 in 52.7%	NA
[20]	Turkey	121	At least mild anxiety in 36.4% using Beck anxiety scale	NA	NA
[21]	Turkey	161 (71.3% consultants)	Anxiety mentioned by 91.3%, mostly from transmission risk to family members (83.1%)	NA	NA
[7]	Turkey	360	DASS-A ≥ 8 in 56.9%; DASS-S ≥ 15 in 43%	DASS-D ≥ 10 in 65%	ISI ≥ 8 in 46.9%
[8]	Spain	328 (108 trainees)	Increased anxiety levels in 58.8%; start of anxiolytic or sleep-inducing treatment in 12.5%	NA	Worsened sleep quality in 53.7%
[22]	New Zealand	57	No reported significant impact on mental health from the COVID-19 lockdown overall: about 30% reported negative impact, while about 37% reported a positive impact		
[23]	Canada	102 residents	Higher anxiety in 56.9%	NA	NA
[24]	India	716 trainees	Increased stress levels in 54.8% during the lockdown	46.5% were unhappy during the lockdown	NA
[25]	Saudi Arabia	108 residents	NA	PHQ-9 ≥ 5 in 92.6%; PHQ-9 ≥ 10 in 49.1%	NA
[26]	Egypt	79 young ophthalmologists and residents	7.6% extremely anxious regarding psychological concerns about the pandemic	NA	NA

# = number of; DASS = Depression, Anxiety and Stress Scale; DASS-A = DASS anxiety scale; DASS-D = DASS depression scale; DASS-S = DASS stress scale; GAD-7 = Generalized Anxiety Disorder-7; HCW = health care workers; ISI = Insomnia Severity Index; NA = not applicable; PHQ-9 = Patient Health Questionnaire-9; PSS-10 = Perceived Stress Scale-1.

## Data Availability

The dataset used in the current study is available from the corresponding author on reasonable request.
